# Effects of Physicians’ Information Giving on Patient Outcomes: a Systematic Review

**DOI:** 10.1007/s11606-021-07044-5

**Published:** 2021-08-05

**Authors:** Hanne C. Lie, Lene K. Juvet, Richard L. Street, Pål Gulbrandsen, Anneli V. Mellblom, Espen Andreas Brembo, Hilde Eide, Lena Heyn, Kristina H. Saltveit, Hilde Strømme, Vibeke Sundling, Eva Turk, Julia Menichetti

**Affiliations:** 1 Department of Behavioral Medicine, Institute of Basic Medical Sciences, Faculty of Medicine, University of Oslo, Oslo, Norway; 2 Centre for Health and Technology, Faculty of Health and Social Sciences, University of South-Eastern Norway, Drammen, Norway; 3 Norvegian Institute of Public Health, Oslo, Norway; 4 Department of Communication, Texas A&M University, College Station, TX USA; 5 Institute of Clinical Medicine, University of Oslo, Oslo, Norway; 6 Health Services Research (HØKH) Centre, Akershus University Hospital, Lørenskog, Norway; 7 Regional Centre for Child and Adolescent Mental Health, Eastern and Southern Norway (RBUP), Oslo, Norway; 8 Library of Medicine and Science, University of Oslo, Oslo, Norway; 9 Department of Optometry, Radiography and Lighting Design, University of South-Eastern Norway, Kongsberg, Norway; 10 Medical Faculty, University of Maribor, Maribor, Slovenia

**Keywords:** systematic review, medical information, medical communication, behavioral change, information recall

## Abstract

**Background:**

Providing diagnostic and treatment information to patients is a core clinical skill, but evidence for the effectiveness of different information-giving strategies is inconsistent. This systematic review aimed to investigate the reported effects of empirically tested communication strategies for providing information on patient-related outcomes: information recall and (health-related) behaviors.

**Methods:**

The databases MEDLINE, Embase, PsycINFO (Ovid), Cochrane Central Register of Controlled Trials, and relevant bibliographies were systematically searched from the inception to April 24, 2020, without restrictions, for articles testing information-giving strategies for physicians (PROSPERO ID: CRD42019115791). Pairs of independent reviewers identified randomized controlled studies with a low risk of selection bias as from the Cochrane risk of bias 2 tool. Main outcomes were grouped into patient information recall and behavioral outcomes (e.g., alcohol consumption, weight loss, participation in screening). Due to high heterogeneity in the data on effects of interventions, these outcomes were descriptively reported, together with studies’, interventions’, and information-giving strategies’ characteristics. PRISMA guidelines were followed.

**Results:**

Seventeen of 9423 articles were included. Eight studies, reporting 10 interventions, assessed patient information recall: mostly conducted in experimental settings and testing a single information-giving strategy. Four of the ten interventions reported significant increase in recall. Nine studies assessed behavioral outcomes, mostly in real-life clinical settings and testing multiple information-giving strategies simultaneously. The heterogeneity in this group of studies was high. Eight of the nine interventions reported a significant positive effect on objectively and subjectively measured patients’ behavioral outcomes.

**Discussion:**

Using specific framing strategies for achieving specific communication goals when providing information to patients appears to have positive effects on information recall and patient health–related behaviors. The heterogeneity observed in this group of studies testifies the need for a more consistent methodological and conceptual agenda when testing medical information-giving strategies.

**Trial Registration:**

PROSPERO registration number: CRD42019115791

**Supplementary Information:**

The online version contains supplementary material available at 10.1007/s11606-021-07044-5.

## INTRODUCTION

According to gold standards of high-quality, modern medical care, patients should be informed about and involved in their care.^1–3^ This patient right is mandated by law in many countries.^4,5^ Patient recall and comprehension of medical information are prerequisites for providing informed consent, making informed treatment decisions, lifestyle, and self-management, and adhering to treatment recommendations.^6,7^ However, medical information is often complex and place great demands on both physicians’ information giving and knowledge translation skills and the patients’ capacity to understand, remember, and ultimately act on the information received.^8^ The process of informing patients involves a dynamic interplay between physicians’ skills in presenting information in a clear, relevant, and actionable way, and patients’ health literacy skills.^3^ This review focuses on physicians’ information exchange practices and associated patient-related behavioral outcomes.

Physicians have a moral and professional obligation to provide high-quality information to patients and secure their comprehension.^4,9^ Although physicians often assume that their explanations and instructions are easy to understand, they are often misunderstood by their patients.^10,11^ Patients commonly forget or misunderstand 40–80% of the information provided by physicians.^11–14^ The personal and societal costs of ineffective information giving are high: non-adherence to treatments^15^, medical errors^16^, longer hospital stays, frequent re-admissions^17^, patient complaints and litigations^18^, poor patient health^19,20^, and healthcare costs.^21^

Effective information giving requires a complex interaction of content, form, and use: speakers formulate *what* (the content) and choose *how* to say something to achieve their goal. Most of the medical literature has focused on the content of the information. Evidence that informational content on its own promotes patient outcomes is poor and, if present, most studies have focused on visual or written information in addition to the medical talk.^21–23^ Little attention has been given to *how* the medical information is provided by physicians during a consultation. Addressing this knowledge gap, we performed an initial scoping review, where we identified a range of strategies for effective information giving for different purposes, i.e., to support patient comprehension, persuade patients, build a relationship, or report facts objectively.^24^ Whether using communication strategies for providing medical information improves patient-related outcomes remains, to the best of our knowledge, unknown.^25^ There is some evidence for an association between general physician communication skills and patient outcomes, but these systematic reviews and/or meta-analyses report on generic communication interventions and show equivocal results.^26–30^ Without evidence for *how* the information is provided by physicians in the medical talk, it is difficult to identify what features of information-giving are associated with better outcomes and how best to design training programs to optimize the effectiveness of the information exchange.

In this systematic review, we describe the reported effects of physicians’ information-giving strategies on patient-related outcomes, as well as the features of these strategies and of the randomized controlled trials (RCTs) testing them.

## METHODS

### Protocol

This systematic review was conducted and reported in accordance with the Preferred Reporting Items for Systematic Review and Meta-analysis (PRISMA) guidelines.^31^ The review protocol is registered in PROSPERO (ID: CRD42019115791).

### Eligibility Criteria

Relevant RCTs were identified through a previously reported scoping review of physicians’ information-giving strategies in the dialog with patients.^24^ RCT studies were eligible for this study if they had a low risk of selection bias assessed with the Risk of Bias 2 (RoB2) tool and if they tested the effect of specified information-giving strategies used by physicians in dialog with patients/analog patients across any medical setting on patient-related outcomes. Studies based on a mix of physicians and other healthcare professionals were included.

Eligible interventions included consultations in which defined strategies for communicating medical information to patients were tested. Eligible comparisons comprised any type of controls.

Outcomes were patient-related, broadly categorized into patient information recall and behavioral outcomes. We excluded outcomes that were assessed in only one eligible study (i.e., satisfaction, quality of life, anxiety, stress, patient’s perceived physician competence). We also excluded trust outcomes because of the scientific debate about the conceptual, methodological, and empirical fragility of trust in the medical relationship^32^, especially in relation to physicians’ information giving.^33^

### Search Strategy and Data Sources

We searched the databases MEDLINE, Embase, PsycINFO (Ovid), and Cochrane Central Register of Controlled Trials from inception to 24 April 2020 without restrictions. We developed the search strategy with an expert medical librarian (HS). Initial search terms were gathered from a set of key articles, then using an iterative process to develop the final search strategy based on relevant key terms and subject headings (Appendix Table 4). We also screened the reference lists of included or relevant articles to retrieve additional references.

### Study Selection

Screening for inclusion in the initial scoping review was conducted independently by five pairs of reviewers. Conflicts were solved by discussion with a third reviewer. Screening for RCTs to be included in this study was performed by three reviewers (J. M., H. C. L., L. K. J.) based on unequivocal low risk of selection bias from RoB2 assessment, reported previously.^24^

### Data Extraction

Data extraction was performed by pairs of researchers. Data on the reported effects of the included interventions on patient-related outcomes were extracted using a predefined document. When different data on the same outcome were reported, we selected the information with greater reliability in terms of type of measure. Authors were contacted to retrieve missing or incomplete data.

Specific data were selected and extracted to describe the studies, Table 1. Details about the information-giving strategies were also extracted from studies, reported in Table 2. Unique information-giving strategies were considered to be the modified minimal units of actions concerning information provision. These were extracted word by word (“Specific message/strategy” in Table 2), organized into strategy types, and strategy types were classified into main categories based on underlying mechanisms of functioning (Table 2, the categorization process is reported elsewhere).^24^

**Table 1:** Characteristics of Information Provision Interventions Assessing Patient Information Recall and Behavioral Outcomes

Author, year, country	Study design	Clinical task	Physicians’ specialty, *n*	Type of patients, *n*	Mean age patients (SD/range); % women
Interventions assessing patient information recall					
Ackermann et al. 2017 (Switzerland) ^34^	RCT	Explaining clinical issues |discharge	Physicians, NR	Analog patients; 234	22 (3.6), 70%
Bennett et al. 2009 (USA) ^35^	RCT	Clarifying informed consent form	Radiologists, 8	Patients undergoing spine injections; 65	NR, NR
Danzi et al. 2018 (Italy) ^36^	Experimental video-vignette study	Explaining treatment under emotions	Physicians, NR	Analog patients |healthy women; 54	25.5 (9.2), 100%
Lehmann et al. 2020a (The Netherlands) ^37^	Experimental video-vignette study	Explaining clinical issues	Oncologists, NR	Analog patients |cancer patients, survivors, healthy; 253	61.3 (11.7), 54%
Lehmann et al. 2020b (The Netherlands) ^38^	Experimental video-vignette study	Explaining clinical issues	Oncologists, NR	Analog patients |cancer patients, survivors, healthy; 148	61.8 (10.1), 50%
Lehmann et al. 2020b (The Netherlands)^38^	Experimental video-vignette study	Explaining clinical issues	Oncologists, NR	Analog patients |cancer patients, survivors, healthy; 148	61.8 (10.1), 50%
Visser et al. 2019 (The Netherlands) ^39^	Experimental video-vignette study	Explaining clinical issues	Physicians, NR	Analog patients |students; 137	21 (2.7), 86%
Visser et al. 2019 (The Netherlands) ^39^	Experimental video-vignette study	Explaining clinical issues	Physicians, NR	Analog patients |students; 136	21 (2.7), 86%
Werner et al. 2013 (Germany) ^40^	RCT	Clarifying informed consent form	Medical students, 30	Analog patients |medical students; 30	25 (4), 57%
Biglino et al. 2015 (UK) ^41^	RCT	Explaining clinical issues	Cardiologists, NR	Parents of children with congenital heart disease; 97	NR, 75%
Interventions assessing patient behavioral outcomes					
Ockene et al. 1999 (USA)^42^	RCT	Improving health behaviors	Mixed (physicians, residents, nurses), 29	High risk drinking; 481	45 (13.4); 37%
Aveyard et al. 2016 (UK) ^43^	RCT	Improving health behaviors	Primary care physicians, 137	Obese; 1882	56 (16.1); 57%
Boguradzka et al. 2014 (Poland) ^44^	RCT	Improving health behaviors	Primary care physicians, 4	Visiting GP for routine medical consultation; 600	NR (50-65); 66%
Grimaldo et al. 2001 (USA) ^45^	RCT	Planning advanced care	Anesthesiologists, 4	Older patients scheduled for elective surgery; 195	72.8 (5.6); 40%
Grover et al. 2007 (Canada) ^46^	RCT	Improving health behaviors	Primary care physicians, 230	High risk cardio patients; 3053	56.3 (8.1); 30%
Kim et al. 2019 (Korea) ^47^	RCT	Improving health behaviors	Cardiologists, NR	Smoking patients with acute coronary syndrome; 66	55.9 (9.0); 3%
Lamb et al. 1994 (USA) ^48^	RCT	Explaining clinical issues |discharge	Mixed (physicians, nurses), NR	Patients with new drugs; 203	53 (NR); 77%
Mazza et al. 2020 (Australia) ^49^	Cluster RCT	Explaining clinical issues	GPs, 57	Sexually active women; 626	NR (16-45); 100%
Saha and Beach 2011 (USA) ^51^	Experimental video-vignette study	Improving health behaviors	Cardiologists, NR	Coronary heart disease patients; 248	58 (10.9); 59%

**Table 2:** Information-Giving Intervention, Strategy(ies), Strategy Type(s), and Strategy Category(ies) Targeted by Each Study

Author, year	Intervention	Specific message/strategy	Strategy type (*N* strategies)	Strategy category	Outcome
Ackermann et al. 2017^34^	Structuring the presentation of discharge information	Structured information, following the structural elements of a book, in which the content is presented in a specific order, from high-level information (e.g., title, table of contents, chapter headings) to detailed, low-level information	Structuring (1)	C	Immediate recall
Bennett et al. 2009^35^	Diagrams added to speech	Showing a set of diagrams illustrating the twelve key points addressed by the informed consent form before signing it	Visualization (1)	C	Recognition
Danzi et al. 2018^36^	Affective communication while delivering bad news	Four supportive statements: “But whatever action we do take, and however that develops, we will continue to take good care of you. We will be with you all the way,” “We will do and will continue to do our very best for you,” “And whatever happens, we will never let you down. You are not facing this on your own,” “I completely understand your reluctance. We’ll look at this decision together carefully and we’ll pay attention to your concerns.”	Emotional-responsiveness (1)	R	Active recall and recognition
Lehmann et al. 2020a ^37^	Tailoring the amount of preferred information	Amount of information tailored to patients’ preferences	Quantity (1)	C	Active recall and recognition
Lehmann et al. 2020b^38^	Affect-oriented, caring communication style	Utterances that validate the patient’s emotional burden and convey understanding (e.g., I can imagine that you’re worried; I understand that this is a tough and uncertain period for you)	Emotional responsiveness (1)	R	Active recall and recognition
Lehmann et al. 2020b^38^	Cognition-oriented communication style with information structuring	Four signs of structuring: verbal signals that introduce a certain topic/agenda, that introduce a summary, that use numeric signals (e.g., first,…second…), and visual signs such as finger/hand signals when counting/using numeric signals	Structuring (1)	C	Active recall and recognition
Visser et al. 2019^39^	Emotion-oriented communication	Emotion-oriented silence (passive style): listen attentively until the patient resumes the conversation	Emotional responsiveness (emotion-oriented silence) (1)	R	Active recall and recognition
Visser et al. 2019^39^	Emotion-oriented communication	Emotion-oriented speech (active style): acknowledging and/or exploring the patient’s emotional expressions, providing empathic and supportive statements	Emotional responsiveness (emotion-oriented speech) (1)	R	Active recall and recognition
Werner et al. 2013^40^	Communication skills training aimed to reduce a layperson’s cognitive load	Assessing what the patient already knows, using easy and understandable language adapted to the patient’s level, active encouragement to ask questions, making use of the available information sheets for medical procedures, reducing the amount of information by clustering the facts (e.g., combining each operative step with its possible complication)	Simplification, structuring, teach-back, visualization (4)	C	Active recall
Biglino et al. 2015^41^	Three-dimensional patient-specific models of cardiac lesion(s) added to speech	Providing a three-dimensional model of the cardiac lesion(s) and discuss it during the appointment	Visualization (1)	C	Change in knowledge
Ockene et al. 1999^42^	Alcohol intervention training with patient-centered counseling approach	Use of nondirective, open-ended questions (e.g., “How do you feel about drinking?” or “How might you go about cutting down?”); the providers were also taught to use patient education materials (i.e., tip sheets) and a goal statement.	Open-ended questions, visualization (2)	C	Alcohol consumption
Aveyard et al. 2016 ^43^	Brief intervention offering referral to a weight management group	Offer of help/referral to change behaviors; ask patients to return	Directivity (1)	P	Weight change
Boguradzka et al. 2014 ^44^	Physicians’ counseling on colonoscopy screening	Standardized discussion with basic information on the disease, rationale for screening and benefits of early treatment and prevention, recommendation to participate in screening, information on screening procedure	Standardization, argumentation (2)	P+O	Participation in screening
Grimaldo et al. 2001^45^	Short information session stressing the importance of patients-proxies’ communication about end-of-life care	Guidelines-driven information; provision of examples regarding cardiopulmonary resuscitation and mechanical ventilation; encouragement to talk with the proxies about end of life wishes	Standardization, accuracy, directivity (3)	P+O	Written durable power of attorney
Grover et al. 2007^46^	Sharing information on future risks for cardiovascular events	Computer printout that displays a patient’s probability of developing coronary disease graphically summarized; ongoing info/feedback	Visualization, repetition (2)	C	Blood lipid levels
Kim et al. 2019^47^	Aversive advice	Three sentences on consequences of dysfunctional behaviors and stress of losses: “Smoking caused your chest pain”; “If you do not stop smoking right now, this pain will come again”; “The next time you feel this pain you will probably die.”	Negative framing (1)	P	Smoking cessation
Lamb et al. 1994^48^	Providing patients with information about potential side effects	Description of potential side effects for new medications, in addition to drug name, purpose, dose	Argumentation (1)	P	Medication side effects
Mazza et al. 2020^49^	Complex intervention providing structured effectiveness-based contraceptive counseling and access to rapid referral	Structured counseling with nonbiased, scripted descriptions of all contraceptives with emphasis on safety and efficacy; recommended return appointment and rapid referral pathway to clinic	Structuring, accuracy, standardization, directivity (4)	C+O+P	Use of contraceptive
Saha and Beach 2011^51^	Patient-centered communication behaviors	Presence of empathic statements, presence of elicitation and validation of patient concerns, more exploration of patient context and individualization of discussion, more rapport building and partnership statements, more patient education, use of lay language, nonverbal behaviors reinforcing verbal behaviors (positive affect showed with voice tone and facial expressions, high attentiveness and presence conveyed through eye contact, nodding, and leaning forward)	Visualization, personalization, emotional responsiveness (3)	C+R	Likelihood of undergoing treatment

*C* cognitive aid strategy (where the strategy had the function of aiding understanding), *O* objectivity-oriented strategy (where the strategy had the function of objectively reporting information), *R* relationship-oriented strategy (where the strategy had the function of building the relationship with the patient), *P* persuasive strategy (where the strategy had the function of persuading the patient to do something)

### Data Analysis

As expected, and described in the study protocol, due to the high heterogeneity of studies, interventions, and outcomes (confirmed also by statistical analyses: *χ*^2^ = 98.62, *p* < 0.001; *I*^2^ = 92% for studies including a behavioral outcome; *χ*^2^ = 11.33, *p* = 0.25; *I*^2^ =21% for studies including information recall as outcome), a meta-analysis with pooled quantitative summary estimates was deemed inappropriate. Therefore, all effects reported, study by study, were qualitatively synthetized, descriptively summarized without summary estimates in tables, and visualized through forest plots obtained with Review Manager version 5.4.1.

## RESULTS

### Overview of Studies

**Figure 1:**
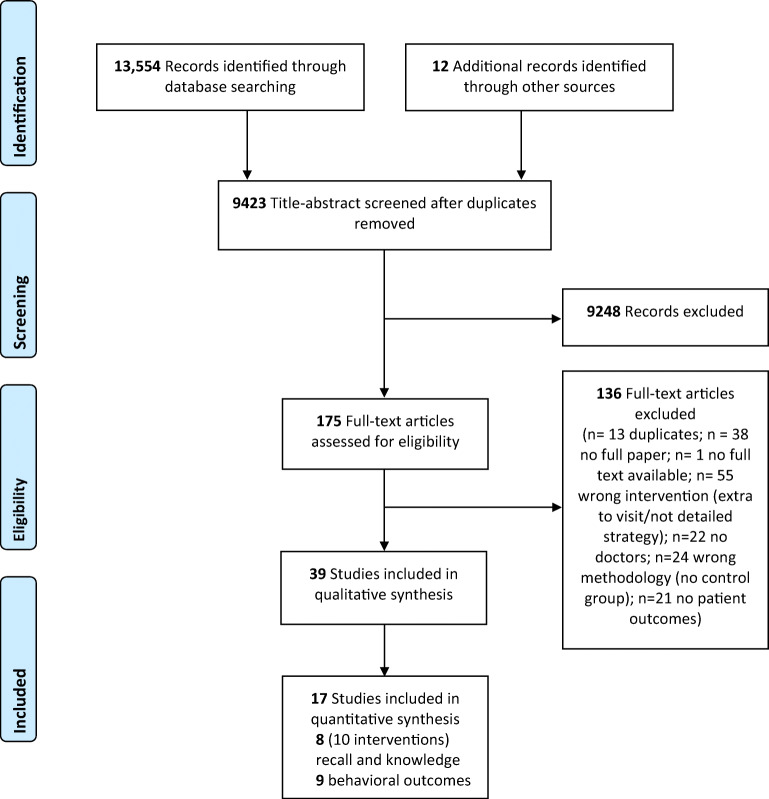
PRISMA Flow chart.

We initially screened 9423 abstracts and 175 full-text articles of which 39 were included in the initial scoping review.^24^ Of these, 17 studies were RCTs with low risk of selection bias and eligible for the systematic review (Fig. 1). Two articles tested two different interventions with different participants each^38,39^, and one of these used one common control group.^39^

Trials were published between 1994 and 2020, and included 8256 patients or analog patients. The average age of patients in the 17 studies was 48 years (*SD* = 17.13). Effects of studies are reported separately for the two main outcomes: information recall and behavioral outcomes.

### Information Recall Studies

The effect of information-giving interventions on recall or knowledge outcomes was investigated in eight of the 17 articles, reporting 10 different interventions. Information recall was most frequently assessed as a combination of free recall and recognition. All recall measures were self-reported and assessed immediately after the intervention. One study assessed self-reported parental knowledge/understanding of the child’s condition before and after the intervention.^41^

#### Characteristics of Studies Assessing Information Recall

The number of patients involved in these studies ranged from 30^40^ to 253^37^ (Table 1). Explaining clinical issues (7 of 10 studies) was the most frequent information provision task. In eight of the 10 interventions, participants were analog patients. Former patients were used in three experimental interventions reported in two articles.^37,38^ Six interventions, reported in four articles, were tested in a fictional experimental setting using video-vignettes^36–39^, while the others were conducted in real life.

All the 10 interventions tested unique groups of strategies, six studies tested cognitive aid strategies, and four relationship-oriented strategies (Table 2). Nine out of 10 studies also tested one single strategy. Overall, the most frequently tested strategies were emotional responsiveness during information-giving dialogs^36,38,39^, information structuring^34,38,40^, and use of visual demonstrations during oral information giving.^34,35,41^

#### Effects of Interventions on Information Recall

**Figure 2:**
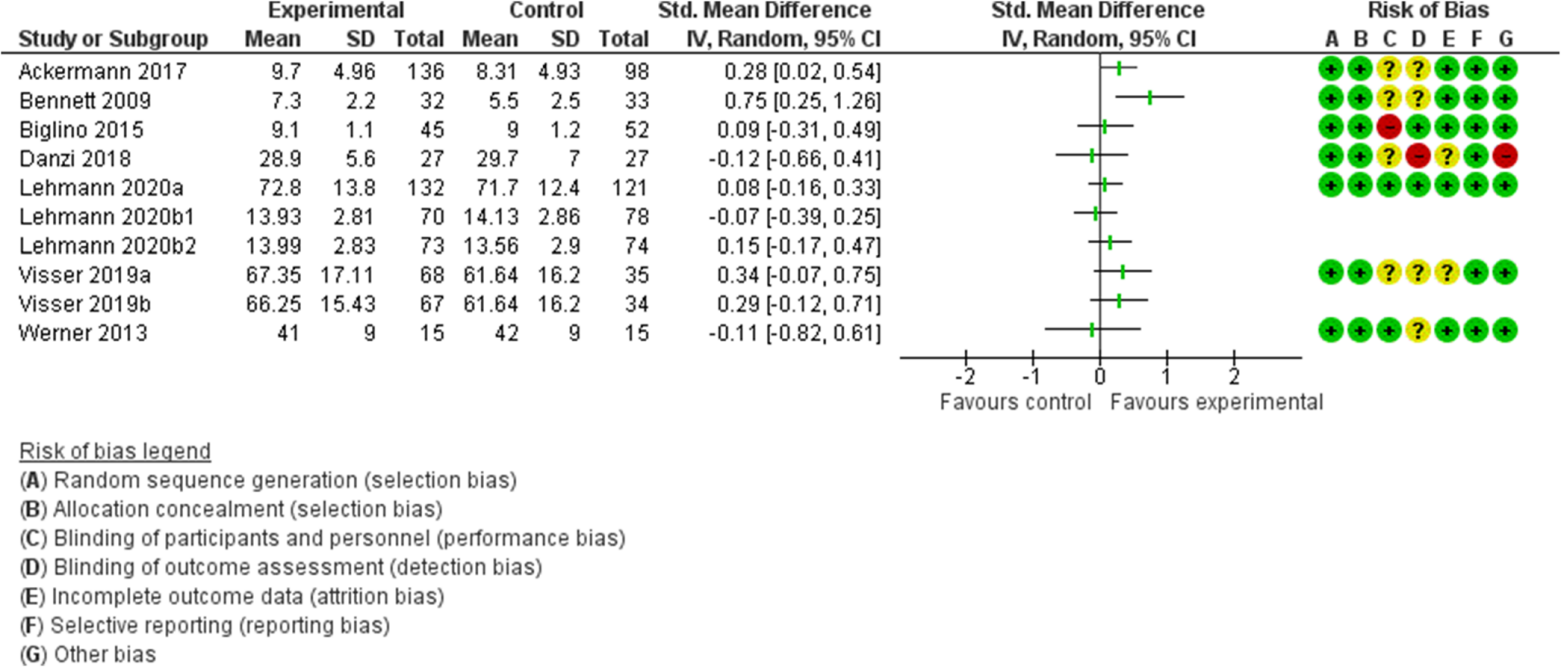
Forest plot of recall or knowledge after information provision interventions.

Seven out of 10 interventions showed a positive effect on information recall, with two studies reporting significant changes^34,35^ and two interventions included in one study reporting significant changes in recognition but not in free recall^39^ (Table 3 and Fig. 2). Ackermann et al.^34^ evaluated the effect of structuring (e.g., akin to a book where high-level information is presented as “title and chapter headings” to low-level information as the text) versus non-structuring of the information given at discharge on the amount of information freely recalled by students (acting as analog patients) with different levels of prior medical knowledge. All 234 participants assigned to the structured discharge consultation significantly increased the number of items recalled (17% increase of recall performance) compared to those receiving non-structured information. The effect was particularly pronounced among those with the least prior medical knowledge (42% increase of recall performance). Bennet et al.^35^ tested a visual method utilizing diagrams to illustrate key points included in the informed consent form on a small sample of 32 patients compared to two control conditions, usual care and “teach-the-teacher” condition where patients are asked to repeat the key points of the informed consent. They found that this visualization strategy significantly increased the number of items recognized by patients compared to the usual care condition, without increasing the average time needed. They did not detect differences between the visualization strategy and the teach-the-teacher strategy, but the latter required more time. Visser et al.^39^ recently compared the effects of oncologists’ emotion-oriented speech and emotion-oriented silence during extensive information-giving sequences on free recall and recognition, compared to giving limited space for emotional disclosure. Both these strategies enhanced recognition but not free recall, with no apparent influence on patient emotional stress level.

**Table 3:** Summary of Results for Intervention and Control Conditions for Each Study

Author, year	Outcome measure (details, range); timing assessment	Strategy type	Type of control	*N* (I)	*N* (C)	Outcome (I)	Outcome (C)	Main effect
Ackermann et al. 2017^34^	Immediate recall (*n* items recalled, 0–28); just after	Structuring	No structuring	136	98	Recalled a mean of 9.7 items (35%) (range = 0–23) (SD = 4.96)	Recalled a mean of 8.31 items (30%) (range = 0–19) (SD = 4.93)	+
Bennett et al. 2009 ^35^	Recognition (multiple choice questionnaire, 0–12); just after	Visualization	Usual care	32	33	mean 7.3 ± SD 2.2 (range 1–10)	mean 5.5 ± SD 2.5 (range 0–10)	+
Danzi et al. 2018 ^36^	Active recall and recognition (8 open-ended, 8 completion, and 8 multiple-choice questions; 0–48); just after	Emotional responsiveness	Same contents, no supportive statements	27	27	mean 28.9 (±5.6) [range 17–38]	mean 29.7 (± 7.0) [range 10–40]	−
Lehmann et al. 2020a^37^	Active recall and recognition (14 open-ended and 14 same multiple-choice questions; 0–27 each); just after	Quantity	Usual care	132	121	For open recall mean 55.9 (SD 17.5); for recognition mean 89.6 (SD 10.1)	For open recall mean 54.9 (SD 14.6); for recognition mean 88.4 (SD 10.1)	−
Lehmann et al. 2020b^38^	Active recall and recognition (14 open-ended and 14 same multiple-choice questions; 0-27 each); just after	Emotional responsiveness	Usual care	70	78	Open recall mean 14.57 (SD 4.06); recognition mean 12.21 (SD 1.56)	Open recall mean 15.94 (SD 4.3); recognition mean 12.32 (SD 1.42)	−
Lehmann et al. 2020b^38^	Active recall and recognition (14 open-ended and 14 same multiple-choice questions; 0–27 each); just after	Structuring	Usual care	74	74	Open recall mean 15.71 (SD 4.1); recognition mean 12.28 (SD 1.57)	Open recall mean 14.87 (SD 4.4); recognition mean 12.26 (SD 1.4)	−
Visser et al. 2019^39^	Active recall and recognition (8 open-ended and 8 same multiple-choice questions; 0–24 each); just after	Emotional responsiveness, passive/emotion-oriented silence	Usual care	68	69	Mean active recall 54.73 (SD 17.2); mean recognition 79.96 (SD 17.02)	Mean active recall 51.9 (SD 16.5); mean recognition 71.37 (SD 15.91)	− for active recall + for recognition
Visser et al. 2019^39^	Active recall and recognition (8 open-ended and 8 same multiple-choice questions; 0–24 each); just after	Emotional responsiveness, active/emotion-oriented speech	Usual care	67	69	Mean active recall 54.52 (SD 15.16); mean recognition 77.98 (SD 15.7)	Mean active recall 51.9 (SD 16.5); mean recognition 71.37 (SD 15.91)	− for active recall + for recognition
Werner et al. 2013^40^	Active recall (n items freely recalled and recorded on a blank sheet of paper); just after	Simplification, structuring, teach-back, visualization	No training	15	15	Mean 41 (SD 9%) after	Mean 42 ± 9% after	−
Biglino et al. 2015^41^	Change in knowledge (self-report questionnaire, 1–10); just after	Visualization	No visual model used during the visit	45	52	Before 7.9±1.6 and after 9.1±1.1	Before mean 8.1± SD 1.7 and after 9.0±1.2	−
Ockene et al. 1999^42^	Alcohol consumption (6-month value minus baseline); 6 months	Open-ended questions, visualization	Usual care	248	233	MD = −6.0 ± SD 11.2	MD = −3.1 ± SD 10.2	+
Aveyard et al. 2016^43^	Weight change (% who lost >5% of weight after 12 months + weight change 0–12 months; 12 months)	Directivity	Advice to change behavior to benefit health	940	942	238 (25%) lost at least 5% of bodyweight; weight change = −2.43 kg	131 (14%) lost at least 5% of bodyweight; weight change = −1.04 kg	+
Boguradzka et al. 2014^44^	Participation in screening; 6 months	Standardization, argumentation	Informational leaflet	300	300	141 (47%) screened	41 (13.7%) screened	+
Grimaldo et al. 2001^45^	Written durable power of attorney completion rates; just after	Standardization, accuracy, directivity	Usual care	97	98	16 (16%) additional patients wrote durable power of attorneys	2 (2%) additional patients wrote durable power of attorneys	+
Grover et al. 2007^46^	Changes in blood lipid levels and the frequency of reaching lipid targets; 12 months	Visualization, repetition	Usual care	1510	1543	835 (55.2%) reach lipid targets	805 (52.2%) reach lipid targets	−
Kim et al. 2019^47^	Smoking cessation rates; 6 months	Negative framing	Usual care	33	33	22 (66.7%) quit smoking at 6 months	10 (30.3%) quit smoking at 6 months	+
Lamb et al. 1994^48^	Patient-reported incidence of side effects for medication; 2–3 weeks	Argumentation	Usual care	104	99	40 (38%) reported side effects	37 (37%) reported side effects	−
Mazza et al. 2020^49^	Use of contraceptive; 2 months	Structuring, accuracy, standardization, directivity	Usual care	248	378	48 (19.3%) with long-acting reversible contraceptive	45 (12.9%) with long-acting reversible contraceptive	+
Saha and Beach 2011 ^51^	Self-reported likelihood of undergoing treatment (4-point scales from definitely to not at all); just after	Visualization, personalization, emotional responsiveness	Low patient-centeredness	134	114	129 (96%) said they would be more likely to undergo treatment	84 (74%) said they would be more likely to undergo treatment	+

*I* intervention, *C* control, *MD* mean difference, *SD* standard deviation, *OD* odds ratio; + = significant effect (<.05); − = no significant effect (>.05)

None of the other interventions reported a statistically significant improvement in information recall. Notably, the most recent study by Lehmann et al. with one of the largest samples in this group of articles (*N*= 148)^38^ tested two of the strategies found to have a positive significant effect in other studies (emotional-responsiveness and information structuring) but reported no improvements in recall. They found that trust may play a conflicting role in recall because enhanced trust decreased recall, and that patients’ personal characteristics (age, gender, education, health literacy) confounded recall outcomes.

### Behavioral Outcomes Studies

Behavioral outcome measures were included in nine of the 17 studies. Behavioral outcomes were assessed objectively in seven and by self-report measures in two studies^48,51^ and included alcohol consumption^42^, weight loss^43^, blood lipid levels^46^, smoking cessation^47^, and treatment-related changes like medication side effects^48^, use of a treatment^49^, or likelihood of undergoing a treatment^51^, participation in screening^44^, and written durable power of attorney.^45^

#### Characteristics of Studies Assessing Behavioral Outcomes

The number of patients involved in the nine studies assessing behavioral outcomes ranged from 66^47^ to 3053^46^ (Table 1). Most studies (8 of 9) were conducted in real-life settings, and one used video-vignettes.^51^ The most frequent clinical task performed was improving health-related behaviors (6 of 9).

In general, each intervention tested multiple information provision strategies (Table 2). The most frequently included strategies were persuasive (5 of 9) and cognitive aid strategies (4 of 9). Two interventions included cognitive aid strategies combined with objectivity-oriented^49^ or relationship-oriented strategies^51^; two interventions included persuasive strategies combined with objectivity-oriented strategies.^44,45^

#### Effect of Interventions on Behavioral Outcomes

**Figure 3:**
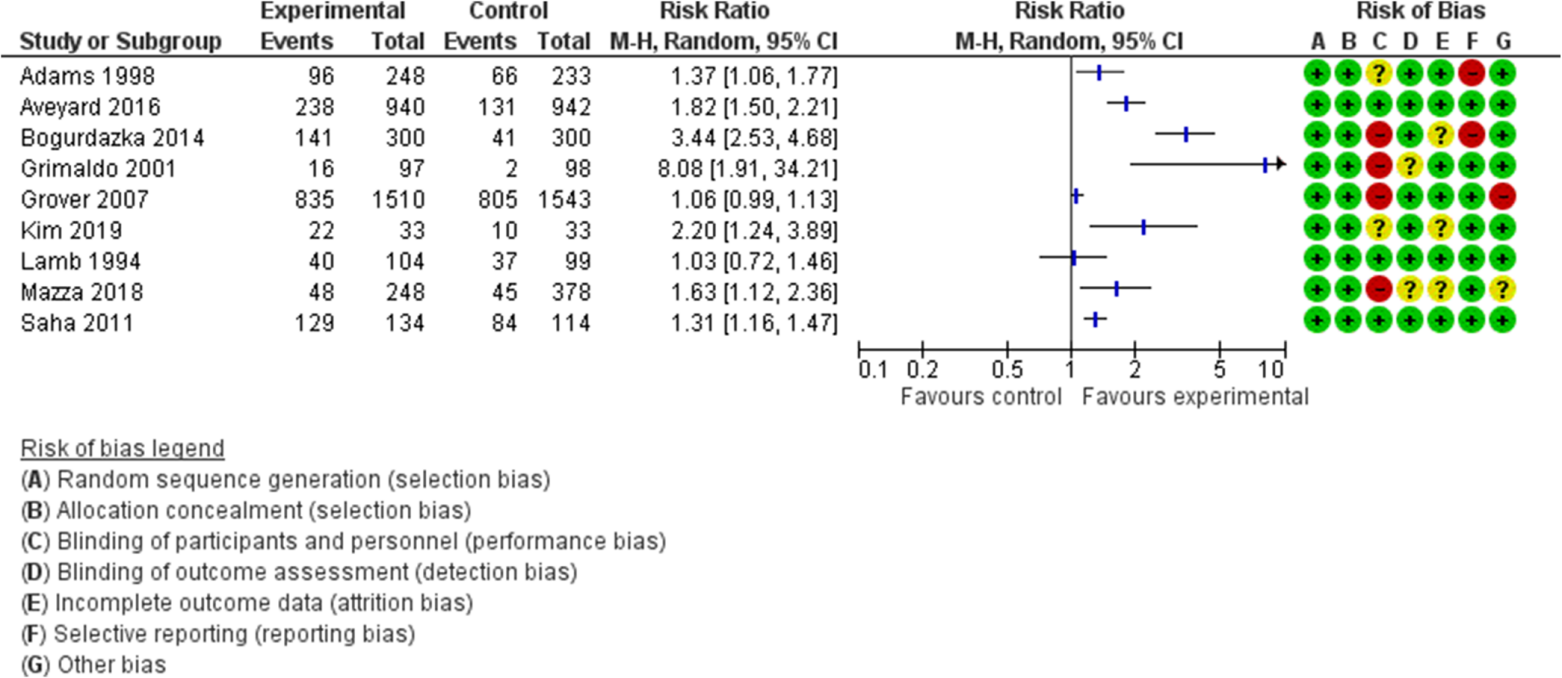
Forest plot of behavioral outcomes after information provision interventions.

Eight of the nine studies reported significant improvements in behavioral outcomes (Table 3 and Fig. 3). All the interventions that included a strategy aimed at persuading patients and influencing their thinking and behavior, by being directive^43,45,49^, providing argumentations^44,48^, or negatively framing the message, reported positive significant improvements on patients’ behaviors. Aveyard et al.^43^, Grimaldo et al.^45^, and Mazza et al.^49^ all tested the effect of a direct recommendation from the doctor to engage in extra-visit activities and of planning a follow-up. These strategies were provided alone^43^ or in combination with other information-giving strategies^45,49^, and led patients to reduce their weight^43^, to write a durable power of attorney after 12 months^45^, or to use contraceptives after 2 months^49^. Both Lamb et al.^48^ and Boguradzka et al.^44^ reported a significant positive impact of providing medical information with full disclosure of benefits and disadvantages (in the case of Boguradzka et al.^44^ together with structured information) on patients’ experienced side effects and participation in screening, respectively. Kim et al.^47^ tested another persuasive strategy for framing an information message: stressing losses and framing the message negatively. The inclusion of three aversive sentences on consequences of smoking led 66.7% of patients to quit smoking after 6 months compared to the 30.3% in usual care.

Both Ockene et al.^42^ and Saha and Beach^51^ tested patient-centered communication strategies in the direction of enhancing patient cognitive processing and understanding of the information (e.g., open-ended questions, lay language, demonstrating with visuals and/or gestures). Saha and Beach^51^ also included information provision strategies supporting the physician-patient relationship like expressing empathy, being responsive to the patients’ concerns, and personalizing the information to make it relevant to the patient’s context. Both studies demonstrated that these interventions improved patients’ health-related behaviors, in the direction of reducing alcohol usage^42^ or increasing the analog patients’ likelihood of undergoing bypass surgery.

The only study that did not report a clear, positive, and significant improvement in patients’ behavioral outcomes (in this case, blood lipid levels, coronary risk, and the frequency of reaching lipid targets) tested the role of repeatedly discussing information on patient’s risk for future cardiovascular events showed in a graphical format with a computer printout.^46^ The findings reported were at the border of significance and Grover et al. discussed that choices in the study may have underestimated the intervention arm.

## Discussion

To the best of our knowledge, this is the first systematic review investigating the effects of information-giving strategies on patient outcomes across different types of medical settings exclusively including RCTs with low risk of bias. This review of 17 RCTs involving 8256 patients provides strong indications that using deliberate communication strategies when providing information can be more effective in improving patient outcomes than not using deliberate strategies. This main finding enriches results from previous systematic reviews showing how physician communication in general^26,28^ and written or visual information outside the medical consultation can improve patient outcomes.^22,23,52,53^ It sheds light on the particular importance of oral information giving, which is routinely used by physicians in their daily practice and do not require additional resources. If oral information provision is deliberately enhanced by specific strategies to frame the information, this may be a powerful tool for improving important cognitive and behavioral outcomes of patients, as well as many other related outcomes.^54^

Four out of 10 studies testing physicians’ information giving strategies on patient information recall reported a positive significant effect. These studies were quite similar in terms of tested strategies, clinical setting, and study design. All but one study testing physicians’ information giving strategies on patients’ behavioral outcomes reported positive significant effects, also on objectively measured outcomes such as weight or blood lipid levels changes. These studies were very heterogeneous including a wide range of strategies and behavioral outcomes’ types, and the findings may have different interpretations and implications.

One possible interpretation involves the extremely different nature of the two considered outcomes and related communication goals (facilitating understanding, changing behaviors), reflected in the use of distinct information framing strategies. In particular, information provision interventions with information recall as outcome mostly tested cognitive aid strategies (like information structuring) in fictitious settings, with the purpose of explaining clinical issues. Some of these studies also tested relationship-oriented strategies (like emotional responsiveness): studies testing relationship-oriented strategies were those showing lowest effects on information recall. One of these studies pointed out the intervening effect of relationship- and trust-related variables on recall^38^, which can potentially explain the reduced impact of this group of studies on recall. The relationship between trust and information recall needs further investigations and may represent a challenge in clinical practice^33^, potentially suggesting a need for physicians to emphasize the importance that patients question their information giving, particularly if patients seem to defer to their authority. On the other hand, for the goal of changing patients’ beliefs or behaviors, persuasive strategies generally yielded strong effects. This supports suggestions provided in a JAMA viewpoint on the essential function of persuasion in medical communication.^55^ Information messages aimed at encouraging patients to engage in certain health behaviors may particularly benefit from deliberate embedding within a persuasion frame. While in this systematic review we focus on explicit persuasive information strategies so that patients become engaged in certain beliefs or behaviors, naturalistic studies have also showed that persuasive attempts can be used in subtle, implicit ways by physicians.^56^ Combined, these results call for a discussion about appropriate and deliberate use of persuasion in physician information giving.

Information recall trials were mostly conducted in fictitious settings and tested unique and consistent strategies, while behavior outcome trials were mainly conducted in real-life settings and tested multiple types of strategies. Real-life studies may introduce more variation in the intervening variables and participants. This may produce greater effects on patient outcomes as patients may find the intervention more relevant to them and/or rely more on the physician’s advice compared to individuals participating as in the shoes of patients or in fictitious scenarios. Previous research has indicated that analog patients are as reliable as actual patients to evaluate physicians’ communication behaviors^57,58^, but this depends on their engagement and by how the scenarios are designed.^59^ Overall, this may indicate the need for a stepwise approach: (1) map behaviors that deserve specific testing, (2) ascertain their potential efficacy in experimental settings, and (3) when variables and mechanisms in play are ascertained, determine how the tested strategy function in real-life settings to produce the desired changes.

Finally, even if the findings reflect information provided for two different communication goals and therefore the strategies used differed substantially, they all have in common the element of information shared by the physician, paired with specific strategies. This may provide some insights about the complex interplay among physicians’ information giving, patient information recall, and patient behaviors. The reported effects on behavioral outcomes may be explained by mechanisms that go beyond the information exchange and involve patients’ perceptions, knowledge, beliefs, attitudes, and intentions to change.^60,61^ Alternatively, patients may prioritize key items of information to remember, those perceived to enable and motivate certain behaviors. Future studies should explore what information patients prioritize as most important to remember, and also what is the minimum number of recalled items necessary to enable engagement in desired behaviors (e.g., participate in screening programs, lifestyle behavior change). Recently, the “learning by doing” pedagogic approach has been stressed, which considers behaviors as facilitators of learning experiences.^62^ Future studies are needed to understand the dynamics between cognitive and behavioral learning processes as a result of different combinations of information contents and strategies, including possible intervening elements such as patient attitudes, perceptions, beliefs, and knowledge.

### Strengths and Limitations

There are several limitations to this study. First, the extent of positive findings in the included studies may be related to publication biases. Second, findings may need to be interpreted separately for studies assessing recall and behavioral outcomes, even if the two outcomes have physicians’ information giving as common denominator. Third, we were able to identify only 17 relevant studies to include with rather small samples, despite all being low-risk RCTs.

Strengths include the rigorous, comprehensive search completed in 2020, and resulted in an overview of a largely unexplored key clinical skill. Findings reflect a strict selection of high-quality articles based on rigorous screening and quality assessment procedures. The study provides a valuable knowledge base for future studies and practical indications for physicians for successfully conveying information to their patients.

## Conclusions

Providing medical information using specific framing strategies appears to improve patient information recall or health-related behaviors. The study offers insights about specific strategies that physicians can deliberately use to frame medical information to reach defined communication goals and improve patient outcomes. Future studies should test the identified strategies with larger samples, in real-life settings to test cognitive aid strategies for securing patient recall, disentangle the complex interplay between different types of strategies concurrently used to deliver similar messages, and teaching courses on information sharing including framing strategies. Finally, future studies should also investigate on the other part of the puzzle, namely to investigate patients’ strategies to make sure physicians understand the information they provide.^63^

## Supplementary Information

ESM 1 (PDF 22.9 kb)
