# Supplementary material for: Effects of Physicians’ Information Giving on Patient Outcomes: a Systematic Review
Source: J Gen Intern Med. 2021 Aug 5;37(3):651–63. doi: 10.1007/s11606-021-07044-5 (PMC8858343; doi:10.1007/s11606-021-07044-5)
Supplement: Supplementary file 1 — (PDF 22.9 kb) [file 11606_2021_7044_MOESM1_ESM.pdf]

Appendices

Appendix 1. Search strategies

Embase 1974 to 2020 April 21 (oemezd)<br>Ovid MEDLINE(R) and Epub Ahead of Print, In-Process & Other Non-Indexed Citations and Daily 1946 to April 21, 2020 (ppez)<br>APA PsycInfo 1987 to April Week 2 2020 (psyf)<br>Date searched: 22 April 2020 (initial search 21 November 2018)<br>Number of hits: 12292 (initial search: 10929)		
1	doctor patient relation/ use oemezd	112137
2	Physician-Patient Relations/ use ppez	71970
3	exp Physicians/ use psyf	36940
4	(physician* or doctor* or surgeon* or general practicioner* or ((medical or surgical or clinical) adj (consultation* or visit*))).ti,ab,kw,kf,id.	1758456
5	or/1-4	1887835
6	(medical information/ or patient guidance/ or patient education/) use oemezd	182573
7	(exp Patient Education as Topic/ or exp Patients/ed or Health Communication/) use ppez	86920
8	Client Education/ use psyf	3629
9	(((medic* or health or patient* or discharge) adj3 (inform* or commuicat* or educat* or advi*)) or (information adj3 (provi* or giv* or fram* or disclos* or deliver* or present*))).ti,ab,kw,kf,id.	1344839
10	or/6-9	1529248
11	(oral communication/ or speech/ or speech articulation/ or speech rate/) use oemezd	55566
12	exp Speech/ use ppez	32157
13	(Oral Communication/ or exp Speech Characteristics/ or Conversation/) use psyf	30836
14	(speech or speak or speaks or speaking or spoke or spoken or say or said or conversation* or dialog* or discuss or discussing or argumentation or talk or talks or talked or talking or tell or telling).ti,ab,kw,kf,id.	1722024
15	or/11-14	1747395
16	5 and 10 and 15	23058
17	(randomis* or randomiz* or randomly or trial or intervention? or effect? or impact? or controlled or control group? or (before adj5 after) or (pre adj5 post) or ((pretest or pre test) and (posttest or post test)) or quasiexperiment* or quasi experiment* or evaluat* or time series or time point? or repeated measur*).ti,ab. use oemezd	12580579
18	(randomis* or randomiz* or randomly or trial or intervention? or effect? or impact? or controlled or control group? or (before adj5 after) or (pre adj5 post) or ((pretest or pre test) and (posttest or post test)) or quasiexperiment* or quasi experiment* or evaluat* or time series or time point? or repeated measur*).ti,ab. use ppez	9822236
19	(("0400" or "0451" or "1800" or "2000").md. or experimental design/ or between groups design/ or quantitative methods/ or quasi experimental methods/ or (randomised or randomized or randomly or controlled or control group? or evaluat* or time series or time point or time points or quasi experiment* or quasiexperiment* or (before adj5 after) or (pre adj5 post) or ((pretest or pre test) and (posttest or post test)) or repeated measur*).ti,ab. or (trial or effect? or impact? or intervention?).ti.) use psyf	2563402

20	17 or 18 or 19	24966217
21	16 and 20	12292
**Cochrane Central Register of Controlled Trials (Cochrane Library, Wiley)**<br>Date searched: 22 April 2020 (initial search 21 November 2018)<br>Number of hits: 1262 (initial search 858)		
#1	[mh ^"Physician-Patient Relations"]	1346
#2	((physician* OR doctor* OR surgeon* OR (general NEXT practicioner*)) OR ((medical OR surgical OR clinical) NEXT (consultation* OR visit*)))	81527
#3	#1 OR #2	81527
#4	([mh "Patient Education as Topic"] OR [mh "Patients"/ed] OR [mh ^"Health Communication"])	8807
#5	(((medic* OR health OR patient* OR discharge) NEAR/3 (inform* OR commuicat* OR educat* OR advi*)) OR (information NEAR/3 (provi* OR giv* OR fram* OR disclos* OR deliver* OR present*)))	77786
#6	#4 OR #5	77914
#7	[mh "Speech"]	754
#8	(speech OR speak OR speaks OR speaking OR spoke OR spoken OR say OR said OR conversation* OR dialog* OR discuss OR discussing OR argumentation OR talk OR talks OR talked OR talking OR tell OR telling)	29711
#9	#7 OR #8	29711
#10	#3 AND #6 AND #9	3165
#11	#10 in Trials	1262
